# Editorial: Microbial fermentation for improved sensory properties and functionality of sustainable foods

**DOI:** 10.3389/fmicb.2024.1387745

**Published:** 2024-03-05

**Authors:** Michela Verni, Sylvester Holt, Carlo Giuseppe Rizzello

**Affiliations:** ^1^Department of Environmental Biology, Sapienza University of Rome, Rome, Italy; ^2^Department of Biology, University of Copenhagen, Copenhagen, Denmark

**Keywords:** fermentation, microbial fermentation, nutrition sustainable food, microbial flavor, yeasts, plant-based

Growing awareness of the health and climate crises has led consumers to reduce meat and alcohol consumption, and government organizations to act on it, driving academic and industry research toward plant-based foods. One of the major challenges in transitioning from animal- to plant-based diets is to replicate the unique sensory characteristics of animal-based foods. Therefore, there is a need for innovation in flavor, texture, and trigeminal sensations to meet the expectations of the growing consumer groups. In this framework, the main objective of this Research Topic was to explore our current understanding of flavor and functionally active microbes in the production of novel foods, with a focus on microbial secondary metabolites.

The volatile flavor compounds of food are closely related to the nature of the microorganisms responsible for fermentation, as ascertained by Wang et al. and Wu et al.. The latter focused on the correlation of dominant microorganisms and the main flavor substances of cigar tobacco leaves during fermentation, whereas the first examined the metabolic improvements in the polyphenolic profile of black tea fermented by *Eurotium crisantum*. The authors provided a comprehensive understanding of the molecular mechanisms affecting the taste and sensory traits of black tea and its related antioxidant potential during the fermentation process (Wang et al.).

While performing their core functions (ethanol production in fermented beverages and carbon dioxide generation in leavened baked goods), yeasts produce several secondary metabolites that contribute significantly to the flavor and aroma of foods and beverages. Higher alcohols and esters deriving from amino acid catabolism are the most abundant of these compounds. Owing to its highly efficient metabolism, *Saccharomyces cerevisiae* is the most preferred yeast species for industrial fermentations, and Li L. et al. reviewed the current literature on the cell-to-cell communication mechanisms in *S. cerevisiae*, studying how its quorum sensing system is essential in stress adaptation, food preservation, and the modulation of metabolites. In another study, indigenous *S. cerevisiae* strains were used to improve the organoleptic profile of Italian Grape Ale beers. Using a multidisciplinary approach, combining results from analyses of the chemical, volatile, and organoleptic profiles of the beers, the authors underlined the relationships between yeast starter and the quality of the final products, thus highlighting the interaction between the strain used and the sensory output (Pietrafesa et al.).

Nevertheless, as opposed to the over-employed and overstudied *S. cerevisiae*, new non-conventional yeast species also offer a very promising route to food and beverage bio-flavoring. For example, while screening non-*Saccharomyces* yeasts for their metabolic performance and oenological properties, Li Y. et al. found that mixed inoculation of the non-*Saccharomyces* yeast strains with *S. cerevisiae* could regulate the volatile aroma characteristics of fermented *Rosa roxburghii* wine, enriching and enhancing its aroma flavor. Non-conventional yeasts are often the key to developing the aroma profile of spontaneous fermented traditional foods and beverages, as is the case of South-road Dark Tea, typical of a Chinese province. It was indeed observed that glycoside hydrolase genes in *Debaryomyces hansenii*, involved in polysaccharide and oligosaccharide degradation as well as catechin transformation, can improve the mellow mouthfeel of South-road Dark Tea (Zou et al.).

In addition to its impact on the sensory properties of food, fermentation can significantly improve the nutritional value of food by introducing new pathways that produce vitamins and micronutrients. This is particularly important for vulnerable groups who may have limited food choices. Fermentation can also increase food functionality by releasing or synthesizing bioactive compounds with functional potential, such as bacteriocins, or providing probiotics and postbiotics. For instance, the fermentation of soybean with *Enterococcus faecium* contributed to flavor development but also the inhibition of *Lysteria monocytogenes* through a bacteriocin produced during fermentation (Kim et al.). Jeon et al., instead, evaluated the prebiotic effect of red ginseng dietary fiber on a *Lactiplantibacillus plantarum* strain. Red ginseng dietary fiber supplementation promoted the probiotic properties of *L. plantarum*, including the production of short chain fatty acids, carbohydrate utilization, attachment to intestinal epithelial cells, and pathogen inhibition.

Improvements with proteins and microbial fermentation are currently in the process of making novel foods a global commercial success. For instance, recent progress in plant-based foods has focused on producing proteins that may lead to umami flavors and precursors that are transformed into savory compounds during the cooking process. The investigation of efficient and cost-effective alternative protein sources is also a topic of interest explored by Li G. et al.. The researchers focused on distillers' dried grains, a co-product of bioethanol production, which are rich in protein. They used an integrated approach that included analyzing the genome of *Paenibacillus pabuli*, assessing *in vitro* enzymatic activities, and conducting solid-state fermentation to assess the suitability of the strain to degrade non-starch polysaccharides.

In conclusion, this Research Topic explored the beneficial effects of microorganisms on food quality and safety, highlighting the correlation between microbial communities and flavor compounds. The reasonable application of beneficial microorganisms is essential for achieving the desired properties, leading to reliable food products and ensuring food quality, safety, and consistency. Although none of the papers published in this Research Topic focused on mimicking the sensory traits that identify animal-based foods, each one of them highlighted specific metabolic pathways that can help steer fermentation processes toward this goal. These pathways could be further explored in future research.

## Author contributions

MV: Writing—original draft, Writing—review & editing. SH: Writing—original draft, Writing—review & editing. CR: Writing—original draft, Writing—review & editing.

